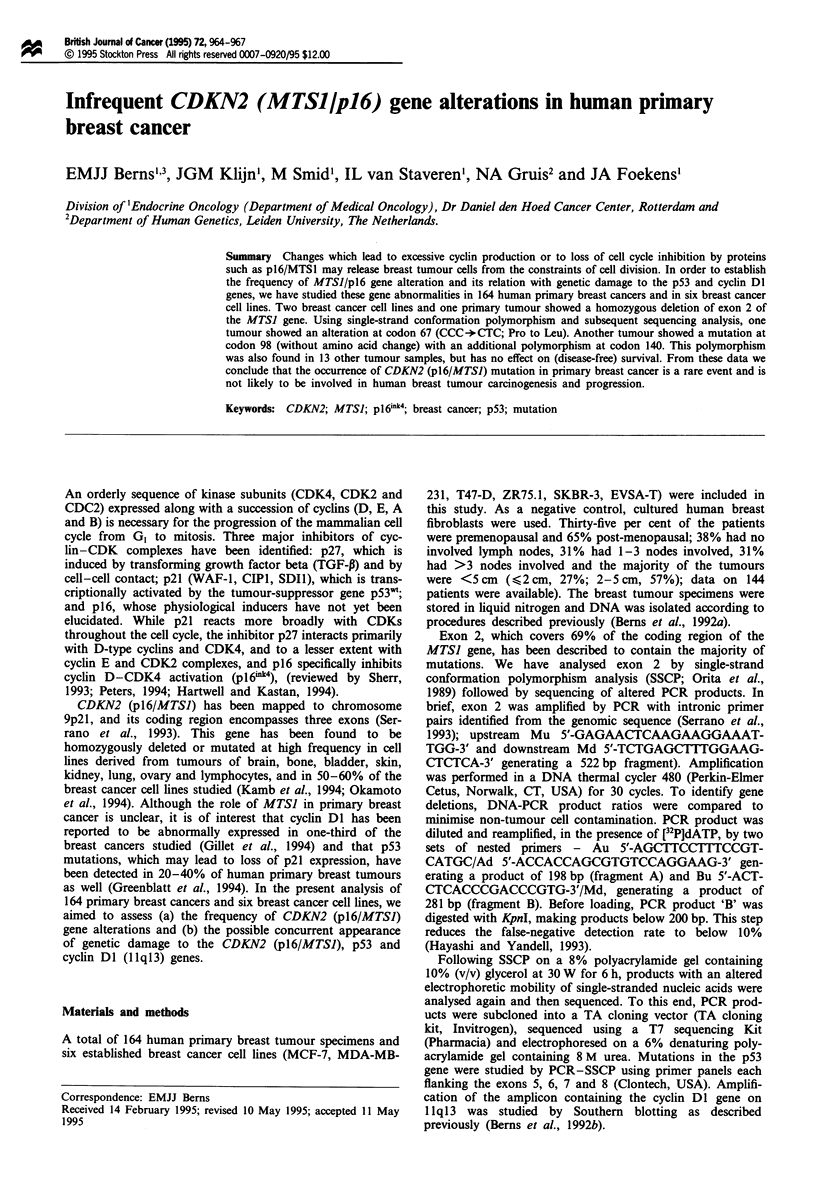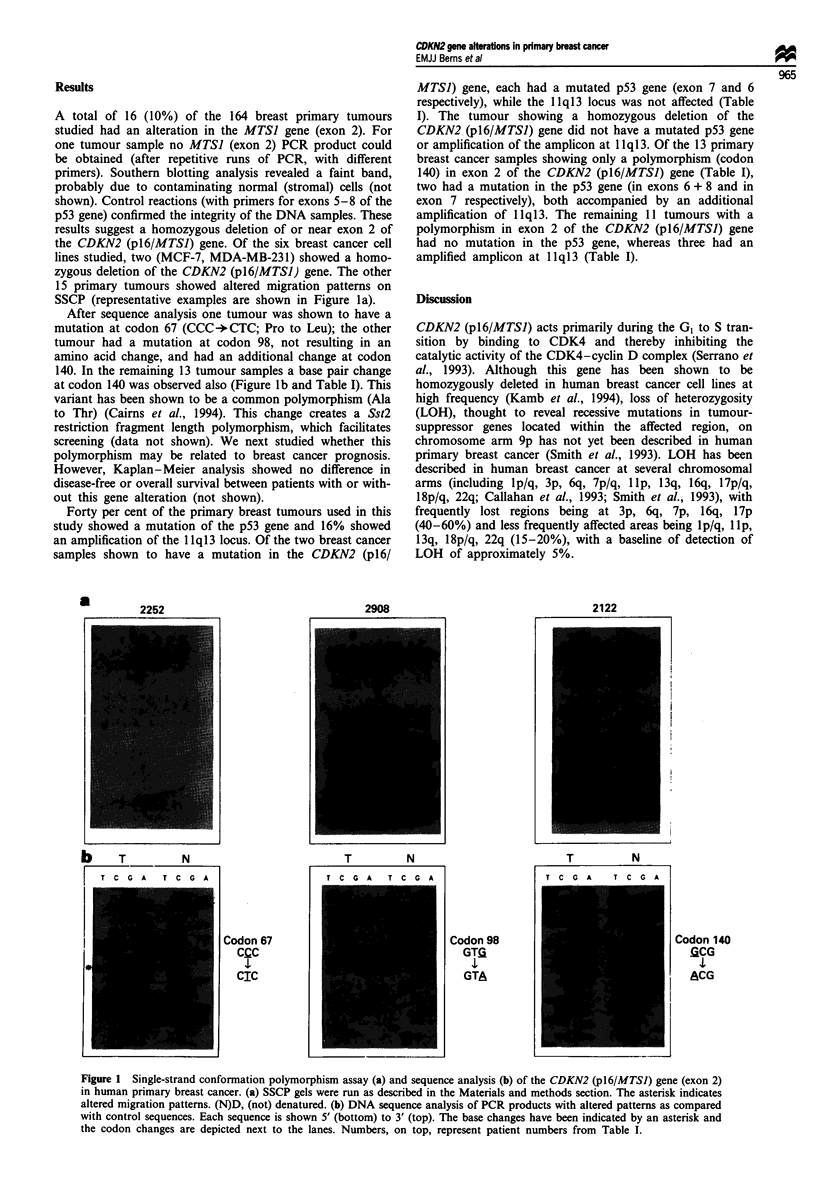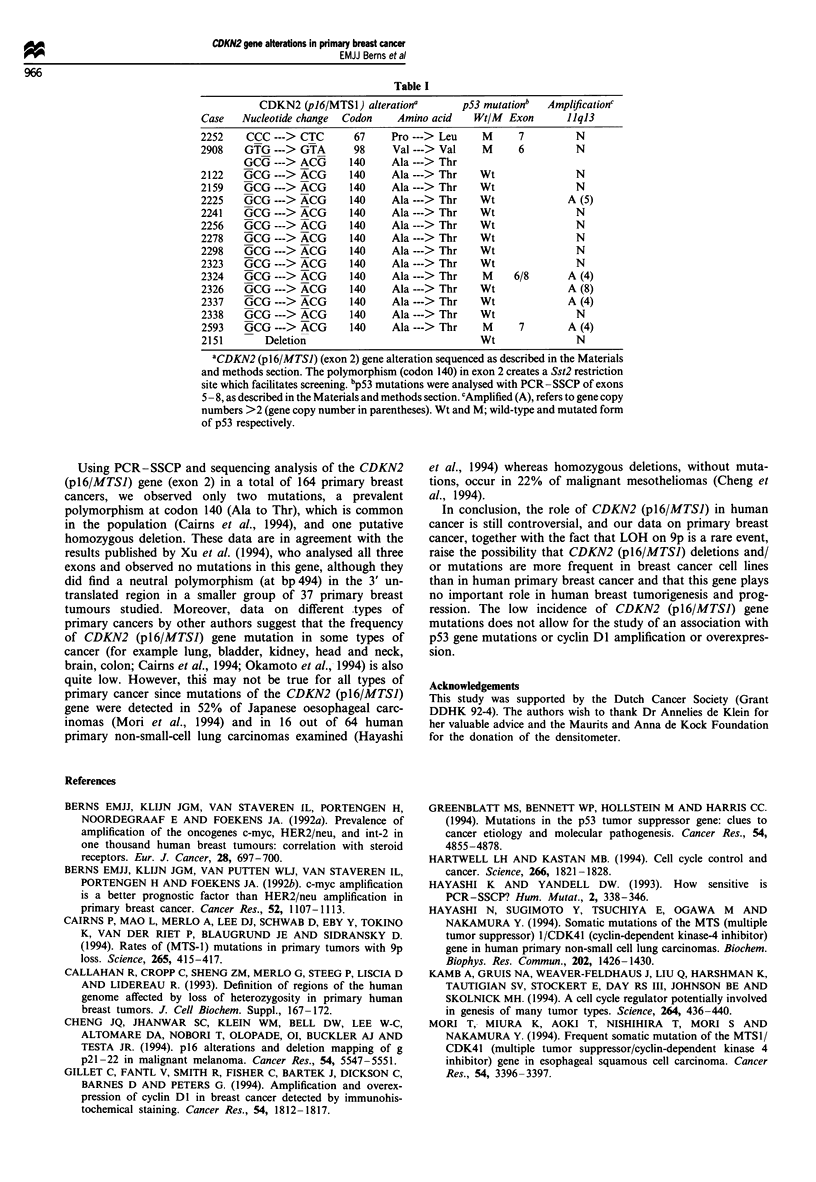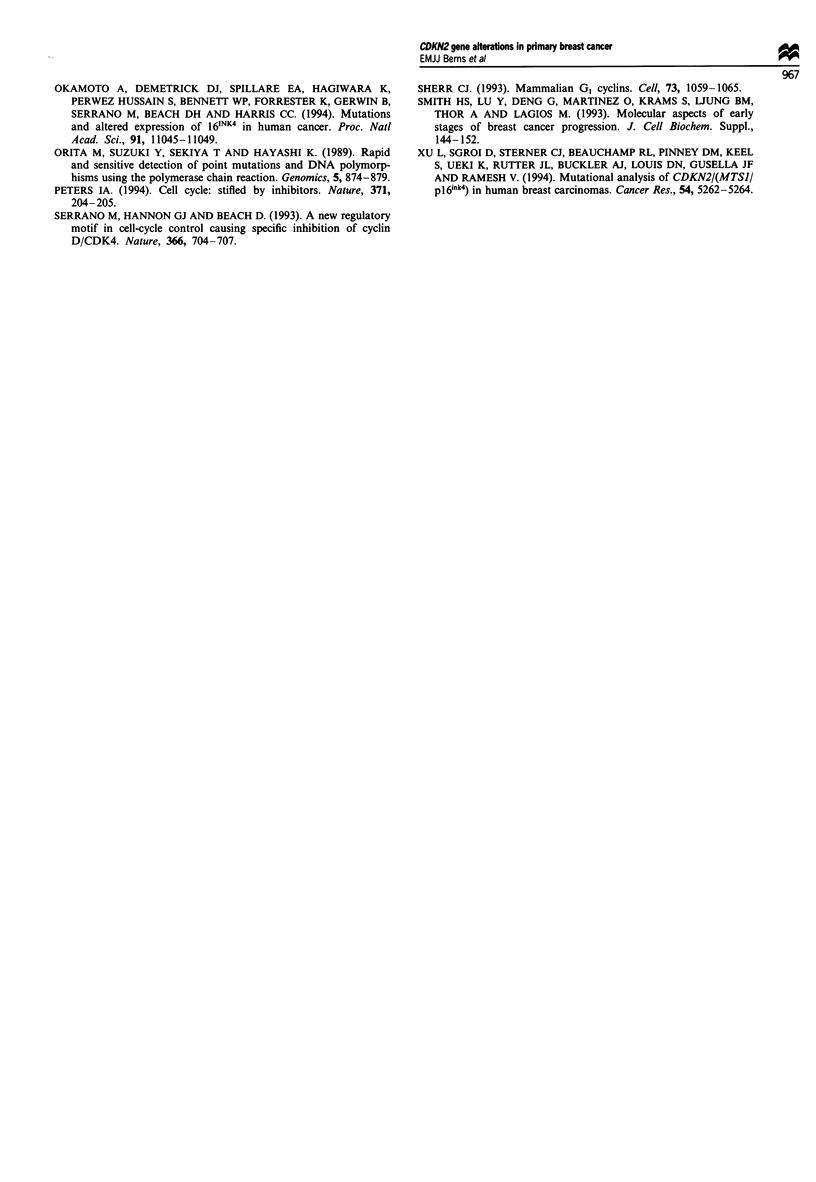# Infrequent CDKN2 (MTS1/p16) gene alterations in human primary breast cancer.

**DOI:** 10.1038/bjc.1995.442

**Published:** 1995-10

**Authors:** E. M. Berns, J. G. Klijn, M. Smid, I. L. van Staveren, N. A. Gruis, J. A. Foekens

**Affiliations:** Division of Endocrine Oncology (Department of Medical Oncology), Dr. Daniel den Hoed Cancer Center, Rotterdam, The Netherlands.

## Abstract

**Images:**


British Journal of Cancer (1995) 72, 964-967
© 1995 Stockton Press All rights reserved 0007-0920/95 $12.00

### Infrequent *CDKN2 (MTS1/p16)* gene alterations in human primary breast cancer

EMJJ Berns[1,3], JGM Klijn[1], M Smid[1], IL van Staveren[1], NA Gruis[2] and JA Foekens[1]

*Division of [1]Endocrine Oncology (Department of Medical Oncology), Dr Daniel den Hoed Cancer Center, Rotterdam and [2]Department of Human Genetics, Leiden University, The Netherlands.*

**Summary** Changes which lead to excessive cyclin production or to loss of cell cycle inhibition by proteins such as p16/MTS1 may release breast tumour cells from the constraints of cell division. In order to establish the frequency of *MTS1*/p16 gene alteration and its relation with genetic damage to the p53 and cyclin D1 genes, we have studied these gene abnormalities in 164 human primary breast cancers and in six breast cancer cell lines. Two breast cancer cell lines and one primary tumour showed a homozygous deletion of exon 2 of the *MTS1* gene. Using single-strand conformation polymorphism and subsequent sequencing analysis, one tumour showed an alteration at codon 67 (CCC→CTC; Pro to Leu). Another tumour showed a mutation at codon 98 (without amino acid change) with an additional polymorphism at codon 140. This polymorphism was also found in 13 other tumour samples, but has no effect on (disease-free) survival. From these data we conclude that the occurrence of *CDKN2* (p16/*MTS1*) mutation in primary breast cancer is a rare event and is not likely to be involved in human breast tumour carcinogenesis and progression.

**Keywords:** *CDKN2*; *MTS1*; p16$^{ink4}$; breast cancer; p53; mutation

An orderly sequence of kinase subunits (CDK4, CDK2 and CDC2) expressed along with a succession of cyclins (D, E, A and B) is necessary for the progression of the mammalian cell cycle from $G_1$ to mitosis. Three major inhibitors of cyclin-CDK complexes have been identified: p27, which is induced by transforming growth factor beta (TGF-$\beta$) and by cell-cell contact; p21 (WAF-1, CIP1, SDI1), which is transcriptionally activated by the tumour-suppressor gene p53$^{wt}$; and p16, whose physiological inducers have not yet been elucidated. While p21 reacts more broadly with CDKs throughout the cell cycle, the inhibitor p27 interacts primarily with D-type cyclins and CDK4, and to a lesser extent with cyclin E and CDK2 complexes, and p16 specifically inhibits cyclin D-CDK4 activation (p16$^{ink4}$), (reviewed by Sherr, 1993; Peters, 1994; Hartwell and Kastan, 1994).

*CDKN2* (p16/*MTS1*) has been mapped to chromosome 9p21, and its coding region encompasses three exons (Serrano *et al.*, 1993). This gene has been found to be homozygously deleted or mutated at high frequency in cell lines derived from tumours of brain, bone, bladder, skin, kidney, lung, ovary and lymphocytes, and in 50-60% of the breast cancer cell lines studied (Kamb *et al.*, 1994; Okamoto *et al.*, 1994). Although the role of *MTS1* in primary breast cancer is unclear, it is of interest that cyclin D1 has been reported to be abnormally expressed in one-third of the breast cancers studied (Gillet *et al.*, 1994) and that p53 mutations, which may lead to loss of p21 expression, have been detected in 20-40% of human primary breast tumours as well (Greenblatt *et al.*, 1994). In the present analysis of 164 primary breast cancers and six breast cancer cell lines, we aimed to assess (a) the frequency of *CDKN2* (p16/*MTS1*) gene alterations and (b) the possible concurrent appearance of genetic damage to the *CDKN2* (p16/*MTS1*), p53 and cyclin D1 (11q13) genes.

### Materials and methods

A total of 164 human primary breast tumour specimens and six established breast cancer cell lines (MCF-7, MDA-MB-231, T47-D, ZR75.1, SKBR-3, EVSA-T) were included in this study. As a negative control, cultured human breast fibroblasts were used. Thirty-five per cent of the patients were premenopausal and 65% post-menopausal; 38% had no involved lymph nodes, 31% had 1-3 nodes involved, 31% had >3 nodes involved and the majority of the tumours were <5 cm (≤2 cm, 27%; 2-5 cm, 57%); data on 144 patients were available). The breast tumour specimens were stored in liquid nitrogen and DNA was isolated according to procedures described previously (Berns *et al.*, 1992*a*).

Exon 2, which covers 69% of the coding region of the *MTS1* gene, has been described to contain the majority of mutations. We have analysed exon 2 by single-strand conformation polymorphism analysis (SSCP; Orita *et al.*, 1989) followed by sequencing of altered PCR products. In brief, exon 2 was amplified by PCR with intronic primer pairs identified from the genomic sequence (Serrano *et al.*, 1993); upstream Mu 5′-GAGAACTCAAGAAGGAAAT-TGG-3′ and downstream Md 5′-TCTGAGCTTTGGAAG-CTCTCA-3′ generating a 522 bp fragment). Amplification was performed in a DNA thermal cycler 480 (Perkin-Elmer Cetus, Norwalk, CT, USA) for 30 cycles. To identify gene deletions, DNA-PCR product ratios were compared to minimise non-tumour cell contamination. PCR product was diluted and reamplified, in the presence of [$^{32}$P]dATP, by two sets of nested primers - Au 5′-AGCTTCCTTTCCGT-CATGC/Ad 5′-ACCACCAGCGTGTCCAGGAAG-3′ generating a product of 198 bp (fragment A) and Bu 5′-ACT-CTCACCCGACCCGTG-3′/Md, generating a product of 281 bp (fragment B). Before loading, PCR product 'B' was digested with *Kpn*I, making products below 200 bp. This step reduces the false-negative detection rate to below 10% (Hayashi and Yandell, 1993).

Following SSCP on a 8% polyacrylamide gel containing 10% (v/v) glycerol at 30 W for 6 h, products with an altered electrophoretic mobility of single-stranded nucleic acids were analysed again and then sequenced. To this end, PCR products were subcloned into a TA cloning vector (TA cloning kit, Invitrogen), sequenced using a T7 sequencing Kit (Pharmacia) and electrophoresed on a 6% denaturing polyacrylamide gel containing 8 M urea. Mutations in the p53 gene were studied by PCR-SSCP using primer panels each flanking the exons 5, 6, 7 and 8 (Clontech, USA). Amplification of the amplicon containing the cyclin D1 gene on 11q13 was studied by Southern blotting as described previously (Berns *et al.*, 1992*b*).

Correspondence: EMJJ Berns
Received 14 February 1995; revised 10 May 1995; accepted 11 May 1995

### Results

A total of 16 (10%) of the 164 breast primary tumours studied had an alteration in the *MTS1* gene (exon 2). For one tumour sample no *MTS1* (exon 2) PCR product could be obtained (after repetitive runs of PCR, with different primers). Southern blotting analysis revealed a faint band, probably due to contaminating normal (stromal) cells (not shown). Control reactions (with primers for exons 5–8 of the p53 gene) confirmed the integrity of the DNA samples. These results suggest a homozygous deletion of or near exon 2 of the *CDKN2* (p16/*MTS1*) gene. Of the six breast cancer cell lines studied, two (MCF-7, MDA-MB-231) showed a homozygous deletion of the *CDKN2* (p16/*MTS1)* gene. The other 15 primary tumours showed altered migration patterns on SSCP (representative examples are shown in Figure 1a).

After sequence analysis one tumour was shown to have a mutation at codon 67 (CCC→CTC; Pro to Leu); the other tumour had a mutation at codon 98, not resulting in an amino acid change, and had an additional change at codon 140. In the remaining 13 tumour samples a base pair change at codon 140 was observed also (Figure 1b and Table I). This variant has been shown to be a common polymorphism (Ala to Thr) (Cairns *et al.*, 1994). This change creates a *Sst*2 restriction fragment length polymorphism, which facilitates screening (data not shown). We next studied whether this polymorphism may be related to breast cancer prognosis. However, Kaplan–Meier analysis showed no difference in disease-free or overall survival between patients with or without this gene alteration (not shown).

Forty per cent of the primary breast tumours used in this study showed a mutation of the p53 gene and 16% showed an amplification of the 11q13 locus. Of the two breast cancer samples shown to have a mutation in the *CDKN2* (p16/*MTS1*) gene, each had a mutated p53 gene (exon 7 and 6 respectively), while the 11q13 locus was not affected (Table I). The tumour showing a homozygous deletion of the *CDKN2* (p16/*MTS1*) gene did not have a mutated p53 gene or amplification of the amplicon at 11q13. Of the 13 primary breast cancer samples showing only a polymorphism (codon 140) in exon 2 of the *CDKN2* (p16/*MTS1*) gene (Table I), two had a mutation in the p53 gene (in exons 6 + 8 and in exon 7 respectively), both accompanied by an additional amplification of 11q13. The remaining 11 tumours with a polymorphism in exon 2 of the *CDKN2* (p16/*MTS1*) gene had no mutation in the p53 gene, whereas three had an amplified amplicon at 11q13 (Table I).

### Discussion

*CDKN2* (p16/*MTS1*) acts primarily during the $G_1$ to S transition by binding to CDK4 and thereby inhibiting the catalytic activity of the CDK4–cyclin D complex (Serrano *et al.*, 1993). Although this gene has been shown to be homozygously deleted in human breast cancer cell lines at high frequency (Kamb *et al.*, 1994), loss of heterozygosity (LOH), thought to reveal recessive mutations in tumour-suppressor genes located within the affected region, on chromosome arm 9p has not yet been described in human primary breast cancer (Smith *et al.*, 1993). LOH has been described in human breast cancer at several chromosomal arms (including 1p/q, 3p, 6q, 7p/q, 11p, 13q, 16q, 17p/q, 18p/q, 22q; Callahan *et al.*, 1993; Smith *et al.*, 1993), with frequently lost regions being at 3p, 6q, 7p, 16q, 17p (40–60%) and less frequently affected areas being 1p/q, 11p, 13q, 18p/q, 22q (15–20%), with a baseline of detection of LOH of approximately 5%.

**Figure 1** Single-strand conformation polymorphism assay (**a**) and sequence analysis (**b**) of the *CDKN2* (p16/*MTS1*) gene (exon 2) in human primary breast cancer. (**a**) SSCP gels were run as described in the Materials and methods section. The asterisk indicates altered migration patterns. (N)D, (not) denatured. (**b**) DNA sequence analysis of PCR products with altered patterns as compared with control sequences. Each sequence is shown 5′ (bottom) to 3′ (top). The base changes have been indicated by an asterisk and the codon changes are depicted next to the lanes. Numbers, on top, represent patient numbers from Table I.

**Table I**

| | CDKN2 (*p16*/MTS1) alteration[a] | | | p53 mutation[b] | | Amplification[c] |
|---|---|---|---|---|---|---|
| *Case* | *Nucleotide change* | *Codon* | *Amino acid* | *Wt/M* | *Exon* | *11q13* |
| 2252 | CCC ---> CTC | 67 | Pro ---> Leu | M | 7 | N |
| 2908 | GTG ---> GTA | 98 | Val ---> Val | M | 6 | N |
| | GCG ---> ACG | 140 | Ala ---> Thr | | | |
| 2122 | GCG ---> ACG | 140 | Ala ---> Thr | Wt | | N |
| 2159 | GCG ---> ACG | 140 | Ala ---> Thr | Wt | | N |
| 2225 | GCG ---> ACG | 140 | Ala ---> Thr | Wt | | A (5) |
| 2241 | GCG ---> ACG | 140 | Ala ---> Thr | Wt | | N |
| 2256 | GCG ---> ACG | 140 | Ala ---> Thr | Wt | | N |
| 2278 | GCG ---> ACG | 140 | Ala ---> Thr | Wt | | N |
| 2298 | GCG ---> ACG | 140 | Ala ---> Thr | Wt | | N |
| 2323 | GCG ---> ACG | 140 | Ala ---> Thr | Wt | | N |
| 2324 | GCG ---> ACG | 140 | Ala ---> Thr | M | 6/8 | A (4) |
| 2326 | GCG ---> ACG | 140 | Ala ---> Thr | Wt | | A (8) |
| 2337 | GCG ---> ACG | 140 | Ala ---> Thr | Wt | | A (4) |
| 2338 | GCG ---> ACG | 140 | Ala ---> Thr | Wt | | N |
| 2593 | GCG ---> ACG | 140 | Ala ---> Thr | M | 7 | A (4) |
| 2151 | Deletion | | | Wt | | N |

[a]*CDKN2* (p16/*MTS1*) (exon 2) gene alteration sequenced as described in the Materials and methods section. The polymorphism (codon 140) in exon 2 creates a *Sst2* restriction site which facilitates screening. [b]p53 mutations were analysed with PCR-SSCP of exons 5-8, as described in the Materials and methods section. [c]Amplified (A), refers to gene copy numbers >2 (gene copy number in parentheses). Wt and M; wild-type and mutated form of p53 respectively.

Using PCR-SSCP and sequencing analysis of the *CDKN2* (p16/*MTS1*) gene (exon 2) in a total of 164 primary breast cancers, we observed only two mutations, a prevalent polymorphism at codon 140 (Ala to Thr), which is common in the population (Cairns *et al.*, 1994), and one putative homozygous deletion. These data are in agreement with the results published by Xu *et al.* (1994), who analysed all three exons and observed no mutations in this gene, although they did find a neutral polymorphism (at bp 494) in the 3′ untranslated region in a smaller group of 37 primary breast tumours studied. Moreover, data on different types of primary cancers by other authors suggest that the frequency of *CDKN2* (p16/*MTS1*) gene mutation in some types of cancer (for example lung, bladder, kidney, head and neck, brain, colon; Cairns *et al.*, 1994; Okamoto *et al.*, 1994) is also quite low. However, this may not be true for all types of primary cancer since mutations of the *CDKN2* (p16/*MTS1*) gene were detected in 52% of Japanese oesophageal carcinomas (Mori *et al.*, 1994) and in 16 out of 64 human primary non-small-cell lung carcinomas examined (Hayashi *et al.*, 1994) whereas homozygous deletions, without mutations, occur in 22% of malignant mesotheliomas (Cheng *et al.*, 1994).

In conclusion, the role of *CDKN2* (p16/*MTS1*) in human cancer is still controversial, and our data on primary breast cancer, together with the fact that LOH on 9p is a rare event, raise the possibility that *CDKN2* (p16/*MTS1*) deletions and/or mutations are more frequent in breast cancer cell lines than in human primary breast cancer and that this gene plays no important role in human breast tumorigenesis and progression. The low incidence of *CDKN2* (p16/*MTS1*) gene mutations does not allow for the study of an association with p53 gene mutations or cyclin D1 amplification or overexpression.

**Acknowledgements**
This study was supported by the Dutch Cancer Society (Grant DDHK 92-4). The authors wish to thank Dr Annelies de Klein for her valuable advice and the Maurits and Anna de Kock Foundation for the donation of the densitometer.

**References**

BERNS EMJJ, KLIJN JGM, VAN STAVEREN IL, PORTENGEN H, NOORDEGRAAF E AND FOEKENS JA. (1992*a*). Prevalence of amplification of the oncogenes c-myc, HER2/neu, and int-2 in one thousand human breast tumours: correlation with steroid receptors. *Eur. J. Cancer*, **28**, 697-700.

BERNS EMJJ, KLIJN JGM, VAN PUTTEN WLJ, VAN STAVEREN IL, PORTENGEN H AND FOEKENS JA. (1992*b*). c-myc amplification is a better prognostic factor than HER2/neu amplification in primary breast cancer. *Cancer Res.*, **52**, 1107-1113.

CAIRNS P, MAO L, MERLO A, LEE DJ, SCHWAB D, EBY Y, TOKINO K, VAN DER RIET P, BLAUGRUND JE AND SIDRANSKY D. (1994). Rates of (MTS-1) mutations in primary tumors with 9p loss. *Science*, **265**, 415-417.

CALLAHAN R, CROPP C, SHENG ZM, MERLO G, STEEG P, LISCIA D AND LIDEREAU R. (1993). Definition of regions of the human genome affected by loss of heterozygosity in primary human breast tumors. *J. Cell Biochem.* Suppl., 167-172.

CHENG JQ, JHANWAR SC, KLEIN WM, BELL DW, LEE W-C, ALTOMARE DA, NOBORI T, OLOPADE, OI, BUCKLER AJ AND TESTA JR. (1994). p16 alterations and deletion mapping of g p21-22 in malignant melanoma. *Cancer Res.*, **54**, 5547-5551.

GILLET C, FANTL V, SMITH R, FISHER C, BARTEK J, DICKSON C, BARNES D AND PETERS G. (1994). Amplification and overexpression of cyclin D1 in breast cancer detected by immunohistochemical staining. *Cancer Res.*, **54**, 1812-1817.

GREENBLATT MS, BENNETT WP, HOLLSTEIN M AND HARRIS CC. (1994). Mutations in the p53 tumor suppressor gene: clues to cancer etiology and molecular pathogenesis. *Cancer Res.*, **54**, 4855-4878.

HARTWELL LH AND KASTAN MB. (1994). Cell cycle control and cancer. *Science*, **266**, 1821-1828.

HAYASHI K AND YANDELL DW. (1993). How sensitive is PCR-SSCP? *Hum. Mutat.*, **2**, 338-346.

HAYASHI N, SUGIMOTO Y, TSUCHIYA E, OGAWA M AND NAKAMURA Y. (1994). Somatic mutations of the MTS (multiple tumor suppressor) 1/CDK41 (cyclin-dependent kinase-4 inhibitor) gene in human primary non-small cell lung carcinomas. *Biochem. Biophys. Res. Commun.*, **202**, 1426-1430.

KAMB A, GRUIS NA, WEAVER-FELDHAUS J, LIU Q, HARSHMAN K, TAUTIGIAN SV, STOCKERT E, DAY RS III, JOHNSON BE AND SKOLNICK MH. (1994). A cell cycle regulator potentially involved in genesis of many tumor types. *Science*, **264**, 436-440.

MORI T, MIURA K, AOKI T, NISHIHIRA T, MORI S AND NAKAMURA Y. (1994). Frequent somatic mutation of the MTS1/CDK41 (multiple tumor suppressor/cyclin-dependent kinase 4 inhibitor) gene in esophageal squamous cell carcinoma. *Cancer Res.*, **54**, 3396-3397.

OKAMOTO A, DEMETRICK DJ, SPILLARE EA, HAGIWARA K, PERWEZ HUSSAIN S, BENNETT WP, FORRESTER K, GERWIN B, SERRANO M, BEACH DH AND HARRIS CC. (1994). Mutations and altered expression of $16^{INK4}$ in human cancer. *Proc. Natl Acad. Sci.*, **91,** 11045–11049.

ORITA M, SUZUKI Y, SEKIYA T AND HAYASHI K. (1989). Rapid and sensitive detection of point mutations and DNA polymorphisms using the polymerase chain reaction. *Genomics*, **5,** 874–879.

PETERS IA. (1994). Cell cycle: stifled by inhibitors. *Nature*, **371,** 204–205.

SERRANO M, HANNON GJ AND BEACH D. (1993). A new regulatory motif in cell-cycle control causing specific inhibition of cyclin D/CDK4. *Nature*, **366,** 704–707.

SHERR CJ. (1993). Mammalian $G_1$ cyclins. *Cell*, **73,** 1059–1065.

SMITH HS, LU Y, DENG G, MARTINEZ O, KRAMS S, LJUNG BM, THOR A AND LAGIOS M. (1993). Molecular aspects of early stages of breast cancer progression. *J. Cell Biochem.* Suppl., 144–152.

XU L, SGROI D, STERNER CJ, BEAUCHAMP RL, PINNEY DM, KEEL S, UEKI K, RUTTER JL, BUCKLER AJ, LOUIS DN, GUSELLA JF AND RAMESH V. (1994). Mutational analysis of *CDKN2*/(*MTS1*/ $p16^{ink4}$) in human breast carcinomas. *Cancer Res.*, **54,** 5262–5264.